# An Embedded Simplified Fuzzy ARTMAP Implemented on a Microcontroller for Food Classification

**DOI:** 10.3390/s130810418

**Published:** 2013-08-13

**Authors:** Eduardo Garcia-Breijo, Jose Garrigues, Luis Gil Sanchez, Nicolas Laguarda-Miro

**Affiliations:** Centro de Reconocimiento Molecular y Desarrollo Tecnológico, Unidad Mixta UPV-UV, Universitat Politècnica de València, Camí de Vera s/n, València E-46022, Spain; E-Mails: jgarrigu@eln.upv.es (J.G.); lgil@eln.upv.es (L.G.S.); nilami@iqn.upv.es (N.L.-M.)

**Keywords:** honey classification, neural networks, fuzzy ARTMAP, microcontroller

## Abstract

In the present study, a portable system based on a microcontroller has been developed to classify different kinds of honeys. In order to do this classification, a Simplified Fuzzy ARTMAP network (SFA) implemented in a microcontroller has been used. Due to memory limits when working with microcontrollers, it is necessary to optimize the use of both program and data memory. Thus, a Graphical User Interface (GUI) for MATLAB^®^ has been developed in order to optimize the necessary parameters to programme the SFA in a microcontroller. The measures have been carried out by potentiometric techniques using a multielectrode made of seven different metals. Next, the neural network has been trained on a PC by means of the GUI in Matlab using the data obtained in the experimental phase. The microcontroller has been programmed with the obtained parameters and then, new samples have been analysed using the portable system in order to test the model. Results are very promising, as an 87.5% recognition rate has been achieved in the training phase, which suggests that this kind of procedures can be successfully used not only for honey classification, but also for many other kinds of food.

## 1. Introduction

Electronic noses and tongues are electronic systems that perform measurements of electrical or optical signals from a set of multiple sensors. Usually, these sensors are not specific because they are not sensitive to any particular physical, chemical or biological parameter, but they are sensitive to a global variation of the environment. Thus, qualitative analyses of different samples with a complex composition can be carried out [1]. Sensors can be very diverse in nature, with emphasis on those that use electrochemical analysis techniques, such as potentiometry [2], voltammetry [3], impedance spectroscopy [4], *etc*.

Some of the most commonly used methods to perform sample classification are those based on Artificial Neural Networks [5–7], named in this way because of their analogy with biological neural systems as they consist of a set of neurons linked together. Adaptive Resonance Theory (ART) is one of the existing neural networks with unsupervised learning methods. This theory was developed in 1976 by Grossberg [8] and he suggested an artificial neural network model whose operation was based on the way the human brain processes information. That is, describing a series of neural network models using supervised and not supervised learning methods to tackle recognition problems and pattern recognition. In 1991, Fuzzy ART [9,10] was also published as a synthesis of Fuzzy Logic Theory. Finally, in 1982, the Fuzzy ARTMAP [11] was published as a supervised version of Fuzzy ART. As the application of these networks was intricate, authors developed their respective algorithmic versions later (1991 and 1992) [12,13].

Since they were created, Fuzzy ARTMAP and Fuzzy ARTMAP Modified Algorithms have been applied to a large number of applications such as electronic nose systems [14,15] and electronic tongues [16,17]. These applications have several advantages: ease of use, low computational cost, as well as transparence and relative simplicity of the implemented algorithms. These algorithms usually work in computer systems based on a PC so that they are able to analyze the data of the obtained measurements.

One of the lines of research to improve electronic tongue systems is the development of electronic systems capable to perform sample analyses *in situ*. Furthermore, autonomous equipment are of interest because they are flexible and easy to use, so they can be used by non-specialized personnel. In order to develop autonomous equipment systems, the incorporation of a neural network in a standalone digital electronic system must be done. The easiest way to create a digital electronic system with these characteristics is by using microcontroller devices because they are cheap, relatively easy to program, information-rich, easy handling and they have low power consumption.

Memory limitation is one of the main features that set microprocessors apart from PC-like systems. Due to this, minimization of the required memory is fundamental when tackling the task to embed a neural network into a microprocessor. In fact, microprocessor memory minimization is defined by the Artificial Neural Networks as they may increase in size when training. This is not a problem when working with a PC but it may become a serious problem when working with microcontrollers. In this way, it is important to optimize the Artificial Neural Network in terms of results and size. This is one of the challenges of this task [17].

The neural network used in this study is a simplified version of the Fuzzy ARTMAP network (Simplified Fuzzy ARTMAP) created by Kasuba [18]. This version simplifies the original algorithm created by Carpenter maintaining good performance. These algorithms are developed to run on mathematical calculation programs such as MATLAB^®^ (The MathWorks, Inc., Natick, MA, USA), through various scripts that simplify their use [17].

In the end, the goal of this paper is the creation of algorithms based on Fuzzy ARTMAP simplified artificial neural networks to be implemented in a microcontroller and the application of these algorithms in the classification of different kinds of honey [19–21]. In order to do this, MATLAB^®^ software programs have been developed by graphical GUI in order to allow the modification of the network properties, and check the size of the memory. The analysis was conducted based on data obtained from potentiometric measurements of the above commented different floral origin honey and the heat treatments. These data have been obtained with a potentiometric electronic tongue system with the described seven electrodes. In addition, an analysis for the electrode selection has been performed, in order to determine the lower number of electrodes to have similar hit rate values than the analyses with the whole electrode array. With this, net properties are improved since the decrease of inputs reduces the memory size.

## 2. Simplified Fuzzy ARTMAP

Most of the aforementioned algorithm applications are implemented in a PC because the memory used is usually big enough to let the algorithms work properly. Problems appear when the algorithms are used in portable systems because low-cost microcontrollers are used in their fabrication and they usually have a limited memory. In this kind of systems, it is necessary to look for the algorithms that fit well in the limited memory of portable systems.

In 1993, Kasuba [18] developed a simplified version of Fuzzy ARTMAP also called SFAM (Simplified Fuzzy ARTMAP). In 2004, Rajasekaran [22] explained the SFAM algorithm based on Kasuba's paper. That year, Aaron Garret (Jacksonville State University) developed a MATLAB^®^ toolbox based on SFAM. The GUI presented in this paper has been developed using this toolbox.

**Figure 1.**
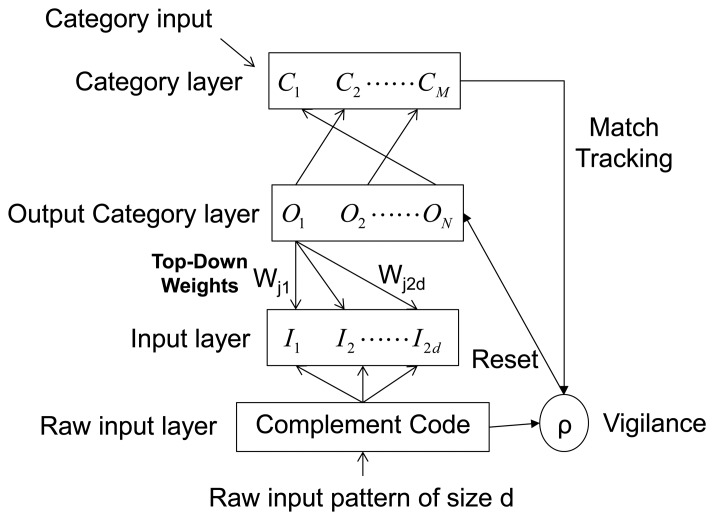
Architecture of the Simplified Fuzzy ARTMAP network.

The network is a step forward for Fuzzy ARTMAP in reducing the computational overhead and architectural redundancy. The model uses simple learning equations with a single user selectable parameter and it can learn every single training pattern within a small number of training iterations. SFAM is faster than FAM and it is easier to program. Figure 1 shows the architecture of SFAM. Vectors {a}, with a number of **d** features, are introduced in the *Complement Code*. There, they are stretched to double the size by adding their complements. The complement code inputs are called {I^a^} and they have a **2d** size. These inputs are introduced into the *Input Layer*. Next, weights (W_j_) from each of the output category nodes O_N_ or subclasses are associated with the input layer vectors. This is the reason why they are called *Top-Down Weights*. Garret designated the *Output Category Layer* as *Mapfield* because of its similar function to the FAM mapfield. The Category Layer (C_M_) contains the labels for the **M** categories or classes that the network has to learn for each one of the input vectors. SFAM network is very sensitive to the absolute magnitudes of the inputs and their fluctuations and it could cause a malfunction of the network. Therefore, it is necessary to normalize the inputs into the same value range.

SFAM operates in two distinct phases: *Supervised Phase or Training Phase* and *Non-Supervised Phase or Test Phase*. A MATLAB^®^-based GUI has been developed for the Supervised Phase as it makes the necessary test parameter calculations easier. The Non-Supervised Phase has been implemented into a microcontroller in order to be used in a portable system.

### 2.1. Non-Supervised Phase: Simplified Fuzzy ARTMAP Graphical User Interface

One of the problems when working with FAM and also SFAM is the size of the Output Category Layer (mapfield) and the Weight matrix that depends on the values chosen for the parameters β and ρ [19]. β is the learning parameter and ρ is the vigilance parameter; both are modified in the Training Phase in order to associate the Entrance Vector to a determinate category. The size of the weight matrix and the mapfield grows if the number of inputs data increases.

Usually there is not an initial criterion to establish the values of these parameters, and several trainings must be done by changing their value in order to find an ideal recognition rate. The Output Category Layer (mapfield) size and the weight matrix size are not usually taken into account when a suitable recognition rate is found because memory size in PC systems is not significant. On the contrary, in microcontroller-based systems it is necessary to verify the sizes of these two data sets and they should be minimum. It can be done by training with different β and ρ values and looking for the best recognition rates in order to select the smallest weight matrix and mapfield sizes.

In order to obtain the maximum recognition rate in the classification for the minimum weight matrix and mapfield, a Graphical User Interface (GUI) has been developed in MATLAB^®^. It is based on the toolbox developed by Garret. By means of this GUI, it is possible to carry out the training considering all the possible values for ß and ρ in order to determine the best classifications and look for the one with the smallest mapfield and data weights size. The idea is to improve the recognition rate in order to implement the network in the microcontroller. In addition, the options of doing a Cross Validation and a variable selection have been implemented into the GUI.

### 2.2. Supervised Phase: Implementation of Simplified Fuzzy ARTMAP Network in the Microcontroller

An aim of the present study is to implement a SFAM neural network in a low-cost microcontroller. The idea is to develop a portable system that could be applied in different fields of the industry. In our case, it is going to be applied in a food industry, such as honey manufacture.

As mentioned before, in low-cost systems, the used microcontrollers usually have a limited size of memory. In these cases it is necessary to optimize the data processing algorithms. In this case, the SFAM contributes with a series of advantages: less memory requirements, rapidity and facility of programming. The information that must be programmed in the microcontroller memory are the Weight Matrix, the Mapfield (Output Category Layer) and the maximum and minimum values of the input vector in order to use them in the normalization of these vectors.

## 3. Materials and Methods

### 3.1. Electrodes and Electronic Systems

Different honey samples have been prepared and measured in order to perform the Fuzzy ARTMAP network analysis and its incorporation into a microcontroller. The data were obtained by an electronic tongue system using potentiometric techniques and it consisted of a set of seven metallic electrodes of different materials. The analyses consisted of measuring four different botanic-origin honey samples. Three of these samples were monofloral honey (citric, rosemary and honeydew) and the fourth one was a mix of different origin honeys (polyfloral). In addition to the botanic origin, three physical treatments usually applied to commercial honey were taken into count: raw, liquation and pasteurization.

Details of these samples and measurements have been published in a previous work [23] showing the measurements made with honey samples from the four different floral origins described above, as well as with three types of heat treatments and four replicates with each of the 12 analyzed samples. We had already used metal electrodes as potentiometric sensors for various applications of food quality control [24,25] and this previous experience let us take the decision to use them in this study too.

Measurements [20,26] have been carried out by using an electronic measuring system consisting of two parts: the array of sensors and the electronic device.

First, the array of sensors has been designed and performed with a set of working electrodes made of different metals. This array has been specifically designed to be immersed in honey samples with a CRISON Ag/AgCl reference electrode (Crison Instruments, SA, Barcelona, Spain) in order to measure the electrical potential spontaneously generated between each working electrode and the reference one. In this case, seven different metallic electrodes have been used. Some of them are made of pure metals as gold, silver and copper in order to study the response of these metal basic sensors. On the other hand, the rest of the electrodes have been chemically treated by electrolysis in order to get specific chemical compounds (AgO_2_, CuO_2_, AgCl and Ag_2_CO_3_) on their surface and determine if they had different properties from the pure metal ones. In addition, some of the electrodes are repeated in the array in order to check the repeatability of the electrochemical response in the samples. In these cases, when repeatability is confirmed, the average values of their measurements are used. In a physical point of view, electrodes were made of metal wires (0.8 mm diameter and 5 cm length), connected to a ribbon cable that sent the electrical signal to the measurement equipment.

Next, the electronic device has been designed and constructed. In this device, the sensors' signals are logged by the microcontroller and an AC converter and data is processed latter in order to work with the SFAM algorithm. In addition, this electronic device has been specifically designed to be portable and able to measure using up to 16 channels simultaneously. So, it allowed a fast analysis phase and let us carry out the experiences in a reasonable period of time.

Moreover, the measurement process has been divided in two stages: the Supervised Phase and the Non-Supervised Phase. In the Supervised Phase, the data were sent to the PC via a RS232 serial communications link in order to use them in the training algorithm with MATLAB^®^ R2010b. The acquisition software was developed using Visual Basic^®^ 6.0 and Microsoft Excel^®^ 2003 software. In the Non-Supervised Phase, samples were measured and the obtained data was stored directly into the microcontroller in order to be used in the embedded neural network.

The Supervised Stage was performed with some of the available samples. At this stage, the network categories are set out and the data is set in an input vector. Next, the coefficients of the algorithm that configure the network are calculated with this data, In the Non-Supervised, the data from new samples are used as inputs, checking whether the outputs of the active network are correct or not.

GUI MATLAB^®^ 2010b running on a PC has been used to train the networks. The computer to be used is determined by its computing power and ease of implementing the algorithms of the neural networks. By contrast, the Non-Supervised Stage is performed entirely in a microcontroller. To this end, the results obtained in the Training Stage are used as the coefficients of the algorithms that are incorporated into the microcontroller program. With this, once the training stage has been accomplished, the developed system can work independently from a PC. In fact, this is one of the key features of the equipment presented in this paper.

### 3.2. Measurement Process

In order to determine the repetitiveness of the measuring system, four measurements were carried out for each honey sample. In this way, the total number of measurements was 48 (four floral origins × three physical treatments × four repetitions). Thirty two of these measurements were carried out for the net Training Phase and the other 16 measurements were developed for the net Supervised Phase by using the integrated net in the microcontroller.

The electrochemical response depends on the measuring electrode and the specific honey sample. It is also important to consider that measurements with such complex chemical samples as ours are not expected to be very repetitive.

Each assay lasted 5 min and consisted of measurement repetitions every 10 s. The reason to carry out recurrent measurements for 5 min is to assure the electrochemical equilibrium between the electrode and the sample was reached and so the measured signal became stabilized. Once the equilibrium was reached, the values of the last ten samples were taken in order to reduce the effect of electrical noise. In this way, a single value was obtained for each one of the seven working electrodes.

### 3.3. Data Analysis

In order to obtain quantitative and complete conclusions from the measurement results, we decided to work with SFAM networks. In our specific case, two SFAM networks were used. The first one tried to determine the floral origin of the honey samples so the network has four outputs: one output for each honey group. The second neural network consists of three outputs, one for each physical treatment.

With the initial data, a matrix of seven columns and 48 rows was created. Each analyzed sample was called: Citrus (C), Rosemary (R), Polyfloral (PF) and Forest (F), and each one of the treatments: Raw (R), liquid (L) and Pasteurized (P). Table 1 shows the different studied samples and their coding.

### 3.4. Training with the Graphical User Interface

#### 3.4.1. Floral Origin Network

In order to start with the Supervised Phase, a GUI Cross Validation has been done. In this specific case, the chosen order was 4. The order 4 Cross Validation is equal to use 75% of the data for training and 25% of the data to do the validation (the complete data matrix was divided into two groups: 24 data points for training and eight for validation). This process is repeated four times in order to obtain the data with the best recognition rate and the smallest mapfield size. With this, the number of samples and the data for training, validation and the total data are calculated and shown in the screen. In addition, target matrices must be generated in order to carry out training and validation and obtain both hit rates and their corresponding mapfield as functions of β and ρ.

In our case, the software runs the training with the SFAM network by using the whole range for β and ρ. The best result was obtained with samples M1, M2 and M5 to M8 for training, and M3 and M4 for validation. In this case, 100% recognition rates and very small mapfield sizes (1–4) were obtained with ρ in the range of [0.1–0.3] and β in the range of [0.7–0.8]. See Table 2.

Finally, the study to select the variables (input electrodes) can be done by GUI. Variable selection methods let us discriminate the variables that influence in the final result and those that do not. As there are several methods, a comparative table has been done with the most use done. The obtained results by different methods are shown in Table 3.

As mentioned above, a Cross Validation with seven electrodes has a 100% recognition rate when validating with samples M3 and M4, with a mapfield of 1 × 4. A 100% recognition rate has also been obtained with the Stepwise PPN method when working with Au, Ag, Cu and CuO_2_ electrodes. In this way, our decision was to combine the training with samples M1, M2 and M5 to M8, and test the model with samples M3 and M4 by using just Au, Ag, Cu and CuO_2_ sensors; then, the values for the new training were [0.1–0.3] for ρ and [0.7–0.8] for β, having increments of 0.05 for both parameters.

Results are shown on the left side of Table 4. As shown, 100% recognition rates have been obtained in all cases with a very small mapfield size. If we compare these results with the ones obtained for the same values for ρ and β but with just one validation sample (results shown at the right side of Table 4), it can be seen that there are the same successful 100% recognition rates but smaller mapfield sizes. This could induce us to believe that these results are better but it is not true in this case because the number of input variables is bigger (seven *vs.* four).

In this way, values for ρ and β parameters can be chosen (e.g., ρ = 0.3 and β = 0.8). With these values, training is done to obtain the definitive values of the weight matrix, the mapfield and maximum/minimum input values in order to program the microcontroller. In addition, GUI also allows us to study the best case if there were different cases with the same recognition rate and minimum mapfield value.

#### 3.4.2. Physical Treatment Network

In this case, the same study as the one in the previous section has been done and it shows the following result: when developing the network to classify samples by physical treatment, a maximum of 83.3% recognition rate has been obtained for ρ = 0.7 and β = 0.3, with a 1 × 15 mapfield and using samples M1 and M2 for validation.

### 3.5. SFAM Non-Supervised Phase in the Microcontroller

The embedded system has been built around a Microchip PIC18F4550 microcontroller. The PIC18F4550 is a PIC18/8-bit family microcontroller and it has 2 KB of RAM and 32 KB of Reprogrammable Flash Memory. The software was coded for the microcontroller and consisted of two main routines:

- The data acquisition system, where the microcontroller reads the data from the A/D converter and processes them in order to obtain the average of each channel.
- The SFAM algorithm that is used in the microcontroller in order to run the Non-Supervised Phase.

These routines were coded in C language (CCS C) and were converted to HEX code using a cross compiler. The HEX file is downloaded into the flash memory of the microcontroller. The routines were programmed in 14,745 bytes of program memory (45% ROM) and 1,820 bytes of data memory (88.8% RAM).

## 4. Results and Discussion

Once the microcontroller was programmed with the optimized Non-Supervised algorithm, four new honey samples were used (M9 to M12) in the Non-Supervised Phase. Table 5 shows the obtained results by the programmed microcontroller in the SFAM Non-Supervised Phase for seven and four electrodes

**Figure 2.**
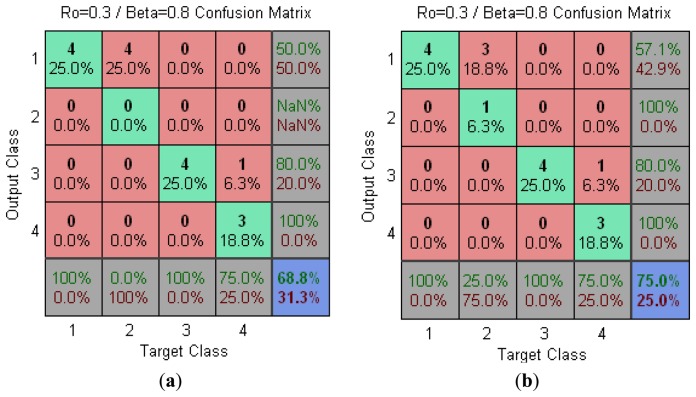
Test data PlotConfusion (seven and four electrodes) with ρ = 0.3 y β = 0.8. (**a**) 7 electrodes. (**b**) 4 electrodes.

Figure 2 shows the confusion matrix for fuzzy ARTMAP. In this case, a recognition rate of 75% has been obtained. It is interesting to highlight that he microcontroller gave a higher recognition rate when working with a lower number of input variables.

## 5. Conclusions

A Graphical User Interface for MATLAB^®^ has been developed in order to optimize the design parameters of a Simplified Fuzzy ARTMAP algorithm in a microcontroller. The Simplified Fuzzy ARTMAP classification algorithm has been implemented both in Supervised and in its Non-Supervised phase in order to classify honeys.

The implementation has been carried out in a portable system based on an 8-bit microcontroller. With the information obtained in the experimental phase, the Simplified Fuzzy ARTMAP network has been trained to obtain the best implementation parameters for microcontroller.

The recognition rate in the Supervised Phase has been 100%. Likewise, a simplification of seven input variables to four has been carried out. With the implemented network in the microcontroller, a test with new samples has been made achieving a recognition rate of 68.8%. This rate has been increased up to 75% by reducing the number of electrodes to four. In addition, this rate could be even higher by increasing the amount of data used in the Supervised Phase. In the end, this study has been carried out with different kinds of honey but the successful obtained results let us predict a similar recognition rate in many other kind of foods.

## Figures and Tables

**Table 1.** Type of honey and theirs assigned classes.

**Honey**	**CR**	**RL**	**PFP**	**FR**	**CL**	**RP**	**PFR**	**FL**	**CP**	**RR**	**PFL**	**FP**	**CR**	**RL**	**PFP**	**FR**
Class	1	2	3	4	1	2	3	4	1	2	3	4	1	2	3	4
Group	M1				M2				M3				M4			

**Honey**	**CL**	**RP**	**PFR**	**FL**	**CP**	**RC**	**PFL**	**FP**	**CR**	**RL**	**FPP**	**FR**	**CL**	**RP**	**PFR**	**FL**

Class	1	2	3	4	1	2	3	4	1	2	3	4	1	2	3	4
Group	M5				M6				M7				M8			

**Table 2.** Recognition rates and mapfield depending on β and ρ for validation with samples M3 and M4.

**Mapfield (1×O)**											**Recognition Rate%**									

**ρ/β**	**0.1**	**0.2**	**0.3**	**0.4**	**0.5**	**0.6**	**0.7**	**0.8**	**0.9**	**1**	**0.1**	**0.2**	**0.3**	**0.4**	**0.5**	**0.6**	**0.7**	**0.8**	**0.9**	**1**
0.1	17	100	6	5	5	5	4	4	6	6	100	75	100	100	100	100	100	100	100	100
0.2	17	100	6	5	5	5	4	4	6	6	100	75	100	100	100	100	100	100	100	100
0.3	17	100	6	5	5	5	4	4	6	6	100	75	100	100	100	100	100	100	100	100
0.4	17	100	6	5	5	5	4	4	6	6	62.5	87.5	87.5	87.5	87.5	87.5	87.5	87.5	87.5	87.5
0.5	17	100	6	5	5	5	4	4	6	6	62.5	87.5	87.5	87.5	87.5	87.5	87.5	87.5	87.5	87.5
0.6	37	100	10	10	9	9	7	8	7	7	75	75	75	75	62.5	75	75	75	87.5	87.5
0.7	31	100	14	14	14	14	13	13	10	8	25	37.5	62.5	50	37.5	50	50	50	50	75
0.8	100	100	20	19	18	21	18	16	15	13	37.5	37.5	75	37.5	50	62.5	62.5	75	50	75
0.9	100	100	23	23	23	23	22	22	22	21	37.5	50	25	37.5	50	37.5	25	25	25	37.5

**Table 3.** Variables selections results [27,28].

**Method**	**Variables Selected**	**Rate**
Forward (3 variables)	Au/Cu/AgCl	------
Forward (4 variables)	Au/Ag/Cu/AgCl	------
Backward (3 variables)	Au/CuO_2_/AgCl	------
Backward (4 variables)	Au/Cu/CuO_2_/AgCl	------
Forward PNN	Au/Ag/Cu	96.875%
Backward PNN	Au/Ag/Cu	96.875%
Stepwise PNN	Au/Ag/Cu/CuO_2_	100%

**Table 4.** Recognition rates and mapfields as a function of ρ and β. Validation with samples M3 and M4 and Au, Ag, Cu and Cu_2_O electrodes.

**ρ/β**	**Validation with M3and M4**						**Validation with M8**					

	**Recognition Rate%**			**Mapfield (1×O)**			**Recognition Rate%**			**Mapfield (1×O)**		

	**0.7**	**0.75**	**0.8**	**0.7**	**0.75**	**0.8**	**0.7**	**0.75**	**0.8**	**0.7**	**0.75**	**0.8**
**0.1**	100	100	100	6	6	6	100	100	100	4	4	4
**0.15**	00	100	100	6	6	6	100	100	100	4	4	4
**0.2**	100	100	100	6	6	6	100	100	100	4	4	4
**0.25**	100	100	100	6	6	6	100	100	100	4	4	4
**0.3**	100	100	100	6	6	6	100	100	100	4	4	4

**Table 5.** Outputs obtained by the Microcontroller with seven and four electrodes.

**Sample**	(**a**) **7 Electrodes**					(**b**) **4 Electrodes**				

	**Class**				**Rate**	**Class**				**Rate**
M9	1	1	3	4	75%	1	2	3	4	100%
M10	1	1	3	3	50%	1	1	3	3	50%
M11	1	1	3	4	75%	1	1	3	4	75%
M12	1	1	3	4	75%	1	1	3	4	75%
